# Two-dimensional electrophoretic comparison of metastatic and non-metastatic human breast tumors using *in vitro *cultured epithelial cells derived from the cancer tissues

**DOI:** 10.1186/1471-2407-8-107

**Published:** 2008-04-16

**Authors:** Jan Vydra, Irena Selicharová, Kateřina Smutná, Miloslav Šanda, Eva Matoušková, Eva Buršíková, Markéta Prchalová, Zuzana Velenská, David Coufal, Jiří Jiráček

**Affiliations:** 1 Department of Oncology, 1st Faculty of Medicine, Charles University Prague, Czech Republic; 2 Institute of Organic Chemistry and Biochemistry, Academy of Sciences of the Czech Republic, Prague, Czech Republic; 3 Institute of Biochemistry and Experimental Oncology, 1st Faculty of Medicine, Charles University Prague, Czech Republic; 4 Institute of Molecular Genetics, Academy of Sciences of the Czech Republic, Prague, Czech Republic; 5 Prague Burn Centre, 3rd Faculty of Medicine, Charles University Prague, Czech Republic; 6 Institute of Pathology 1st Faculty of Medicine, Charles University Prague and General Teaching Hospital, Prague, Czech Republic; 7 Institute of Computer Science, Academy of Sciences of the Czech Republic, Prague, Czech Republic

## Abstract

**Background:**

Breast carcinomas represent a heterogeneous group of tumors diverse in behavior, outcome, and response to therapy. Identification of proteins resembling the tumor biology can improve the diagnosis, prediction, treatment selection, and targeting of therapy. Since the beginning of the post-genomic era, the focus of molecular biology gradually moved from genomes to proteins and proteomes and to their functionality. Proteomics can potentially capture dynamic changes in protein expression integrating both genetic and epigenetic influences.

**Methods:**

We prepared primary cultures of epithelial cells from 23 breast cancer tissue samples and performed comparative proteomic analysis. Seven patients developed distant metastases within three-year follow-up. These samples were included into a metastase-positive group, the others formed a metastase-negative group. Two-dimensional electrophoretical (2-DE) gels in pH range 4–7 were prepared. Spot densities in 2-DE protein maps were subjected to statistical analyses (R/maanova package) and data-mining analysis (GUHA). For identification of proteins in selected spots, liquid chromatography-tandem mass spectrometry (LC-MS/MS) was employed.

**Results:**

Three protein spots were significantly altered between the metastatic and non-metastatic groups. The correlations were proven at the 0.05 significance level. Nucleophosmin was increased in the group with metastases. The levels of 2,3-trans-enoyl-CoA isomerase and glutathione peroxidase 1 were decreased.

**Conclusion:**

We have performed an extensive proteomic study of mammary epithelial cells from breast cancer patients. We have found differentially expressed proteins between the samples from metastase-positive and metastase-negative patient groups.

## Background

Breast cancer is the most common cancer affecting women worldwide. Human breast carcinomas represent a heterogeneous group of tumors diverse in behavior, outcome, and response to therapy. Despite tremendous advances in screening, diagnosis, and treatment, causes of this disease remain elusive and complex.

It has been hypothesized that the clinical and genetic heterogeneity of breast cancer is a result of activation of different oncogenes or loss of different tumor suppressor genes in specific stem/progenitor cells [1]. The genetic and immunohistochemical analysis led to further clasification of human breast cacinomas as basal or luminal according to their cell type origin. To date, five types of breast carcinomas have been recognized according to the molecular genetics profiling [2,3].

The nature of molecular changes varies between breast tumors and determines the characteristics of the disease. Current research priority is to develop methods to identify the most informative molecular changes, also known as disease markers. Thus the treatment strategy could be optimized and individualized using molecular-biological properties of the patient's tumor cells.

At present, several prognostic and predictive factors are commonly used in the breast carcinoma treatment. They include clinical factors such as tumor size, stage and histological type, histological grade, number and scale of regional lymph node involvement, hormone-receptor levels (ER, PR), HER-2/neu expression level and nuclear DNA ploidy. The significance of these factors has been clearly determined and together with the clinical state of the patient they are the main determinants in the process of selection of treatment modality [4]. Despite the research and treatment advances, the outcome of patients is still often poor. Clearly, there is a critical need to find new molecular parameters not only for detection, but also for classification and treatment of the breast cancer.

Proteomics is a rapidly developing field that can explore the heterogeneity of breast cancer and supplement the wealth of information gained from genomics. Breast cancer is one of the most studied cancers in proteomics. Studies investigating differential expression of proteins between normal and breast cancer cells revealed changes in the composition of cytoskeletal elements such as cytokeratin distribution and tropomyosin expression, the differential distribution of molecular chaperones (heat shock protein family members, protein folding enzymes, 14-3-3 σ) has been described together with elevated levels of glycolytic enzymes (aldolase, glyceraldehyde dehydrogenase) [5,6]. Roles of lysozomal proteases (cathepsin D, cathepsin B) and matrix metalloproteases (MMPs) in the breast cancer development and progression have been explored [7].

However, proteomic analysis of larger amounts of clinical samples is so far a challenge [8]. Two-dimensional gel electrophoresis (2-DE) facilitates the separation of proteins from highly complex protein mixtures and has become a central method in proteomics in recent years. Unfortunately, the 2-DE methodology remains labor intensive and also the subsequent gel analysis is difficult. Although the 2-DE processing softwares are continuously developing, their full automation is immense [9,10]. The methodology also puts demands on sample amount and composition. Selection of the most convenient samples containing sufficient amount of proteins suitable for 2-DE proteomic analyses is of crucial importance. Whereas differential proteomic analysis of breast tissue biopsies is complicated due to heterogeneity of cellular phenotypes contained in the sample [11], cells in culture represent a homogenous system, which can be to a certain extent defined and specifically altered.

Optimized feeder layer technique was adapted for cultivation of mammary gland epithelial cells [12]. Successful *in vitro *expansion of luminal cells together with myoepithelial cells in heterogeneous populations of human breast epithelial cells was achieved. It is assumed that among the bulk of cells forming the body of the tumor only a few drives the tumor outgrow. They are supposed to be derived from the so-called stem or progenitor cells [13,14]. Recently, we have characterized a new cell line, EM-G3, possessing some characteristics of putative breast progenitor cells. The cell line was established from the primary culture of breast cancer biopsy sample using the optimized feeder layer technique [15,16]. We believe that our method of temporal *in vitro *propagation of cells from breast cancer tissues could partially lead to the selection of cells relatively close to putative tumor stem cells [12,17]. We performed the 2-DE protein analysis of malignant breast cancer cells cultivated from tissues of different patients in various stages of breast cancer. We tried to find association among possible variations in the expression of proteins and clinical outcome of breast cancer patients. R/computing environment was used to perform statistical analyses [18]. Namely, the analyses based on the R/maanova package [19] have been performed. We further employed the data-mining technique GUHA (General Unary Hypothesis Automaton) to reveal possible relations among protein spots and their impact on clinical image [20]. The GUHA is a method of exploratory data analysis with logical and statistical backgrounds. It automatically formulates and tests a huge amount of hypotheses on relations in data and reveals the "interesting" ones. Some potential candidates for protein markers ensue from these trials.

## Methods

### Patients

The samples were obtained in the years 1999 – 2002 from women who underwent partial breast resection or radical mastectomy at the General Faculty Hospital in Prague. Patients were chosen unselectively at the time of operation. The patient's written informed consent approved by the Ethical committee of the General Faculty Hospital in Prague was obtained prior to surgery. The morphology of tumors was determined and immunocytochemical staining for hormonal receptors (ER, PR), HER 2/neu and antigen Ki67 was performed. The patients were treated according to the stage-adjusted therapeutic standards. We estimated the clinical outcome of the patients. The patients with follow-up at least three years were chosen for further analysis. The patients were divided into two groups: distant metastase-free after three years and patients with proven distant metastases.

### Immunohistochemistry

Paraffin sections 5 μm from formalin-fixed tissues were used. The tissue sections were incubated with primary antibodies ER, Dako (Glostrup, Denmark), clone 1D5, dilution 1 : 100; PR, Novocastra (Newcastle, UK) clone 16, dilution 1 : 100; Ki67, Novocastra (Newcastle, UK) clone MIB-1, dilution 1 : 50. Immunodetection was performed with the universal immuno-peroxidase polymer Histofine, Nichirei Biosciences INC (Tokyo, Japan). Detection of HER 2/neu was performed using HercepTest TM assay detection system, Dako (Glostrup, Denmark). Five percent 3,3'-diaminobenzidine tetrahydrochloride chromogen solution was used for visualization. Positive and negative controls were included in each run of slides.

### Cell cultures

Primary cell cultures were isolated from biopsies of human breast carcinomas. The cells were cultured by the 3T3 feeder-layer technique [12,17].

The cells in the second or third passage were grown to confluence, harvested and stored in liquid nitrogen in the culture medium containing 10% dimethylsulfoxid and 20% of bovine serum. The cells designated for 2-DE analyses were thawed, seeded, cultivated to confluent layers and harvested as described in Selicharova et al. [15]. Out of 120 cultivated samples, primary cultures from tumor tissue of 23 patients were suitable for further 2-DE based analysis because of a sufficient amount of cultivated cells (about five millions).

### Two-dimensional gel electrophoresis

The cell lysate (70 μg of proteins) in rehydration buffer composed of 7 M urea, 2 M thiourea, 4% (w/v) CHAPS, 50 mM DTT, 0.8% (v/v) ampholytes (pH 3–10) was applied to 18 cm linear IPG strips pH 4–7, GE Healthcare (Uppsala, Sweden). 2-DE was performed exactly as described [15]. Briefly, the IEF of rehydrated strips was performed with stepwise increasing voltage as follows: 250 V for 1 h, 500 V for 1 h, 1000 V for 2 h and 10,000 V for the time period necessary to reach 70,000 Vh in total. The focused strips were equilibrated for 30 min in the solution containing 6 M urea, 20% (v/v) glycerol, 2% (w/v) SDS, 0.05 M Tris/HCl pH 8.8 and 2% (v/v) DTT with traces of Bromphenol Blue. Then free thiol groups were alkylated for 30 min in the same solution containing 2.5% (w/v) iodoacetamide instead of DTT. The SDS-PAGE on gradient gels (8–16%, 4% stacking gel, 19 × 22 cm) was performed in 0.025 M Tris/0.192 M Glycine with 0.1% (w/v) SDS running buffer for 1 h at 16 mA and for about 9 h at 24 mA per gel till the Bromphenol Blue line has reached the bottom of the gel. Three silver-stained analytical gels were prepared from each sample. All the common chemicals were from Sigma (St. Louis, USA) and Fluka (Buchs, Switzerland).

### Image analysis

Gels were scanned by a GS-800 Calibrated Densitometer, Bio-Rad (Hercules, CA) at 700 dpi resolution. The images were further processed by PDQuest Advaced 8.0.1 2D Gel Analysis Software, Bio-Rad (Hercules, CA). For computational purposes the file size was reduced to 50% and the images were cropped to frame the same clusters of spots. One or two representative gels per each cell population were used to create a match-set. Spots were detected and matched automatically to a master gel selected by the software. The spot detection and matching were edited manually. The spot boundary tool was applied to detect large spots. The patterns in sections of the gels in appropriate magnification were checked and spots were added manually to the master gel to allow matching unique spots present in the individual gels. The spot quantity table containing all matched spots was generated. The quantity of missing spots was estimated by the software. The means of logarithmic ratios method was used for normalization. The mean of log ratios method of normalization calculates the normalization factor of a gel by calculating the mean of all log ratios (log spot quantity of gel/log spot quantity of master gel) of all matched spots (master gel – gel). The quantity table was exported to a spreadsheet .xls file and submitted to statistical analyses (Additional data file 1).

### Statistical analysis and data mining – GUHA (General Unary Hypothesis Automaton)

Independent statistical tests were performed using R/computing environment in version 2.6.0 [18] and by adapting R/maanova package version 1.8.0. [19] which has been designed for processing microarray data. It implements sample shuffling. R/maanova provides a permutation method to calculate the nominal permutation p-values for each gene (i.e. spot intensity) using Fs test statistics. Because of multiple testing, the p-values were adjusted to false discovery rate [21].

The relations among spot intensities and clinical image were analyzed on the basis of data-mining technique GUHA [20]. The analyzed data were stored in a source database in the form of a table of n rows (objects = 2-DE gels) and m columns (variables = spot intensities). The variables were dichotomized. Each variable was categorized. Categories were actually subsets of ranges of variables given by cut points. The category was evaluated as 1 if the value of a variable dropped within a subset given by the respective cut point otherwise it was evaluated as 0. The settings of cut points were based on the specification and behavior of an impurity function [22]. In our application we employed the entropy function as impurity function for each intensity variable classified with respect to the clinical variable metastases. The impurity function takes its minimum if all objects are classified as 0 or 1. The maximum is reached if roughly a 50/50 mixture of classes is present in the group. The idea is to split the original group into two sub-groups in such a way that impurity decreases in a maximal way, i.e., that the sum of impurities of sub-groups is minimized. We identified optimal splits and corresponding optimal cut points by a script in MATLAB [23]. The cut points then enabled us to categorize spot intensities. A GUHA hypothesis is determined by the ordered pair of cedents (*antecedent *(A) and *succedent *(S)) and by a *quantifier*. Cedents are Boolean conjunctions formed from individual categories. The length of a cedent is given by the number of categories forming the conjunction. A cedent of length = 1 corresponds to a single category (simple cedent). Cedents of length > 1 are called compound cedents. For a given object a cedent can be evaluated as 1 (true) or 0 (false). The evaluation stems from the evaluation of single categories forming the cedent and rules for Boolean conjunction. For a given pair of cedents, we can construct a corresponding contingency table by evaluating cedents for all objects in the database and then perform statistical tests on this table. The employed quantifier determines the type of test. We used the Fisher quantifier corresponding to Fisher's exact test. A hypothesis is formally written A ~S, and if it is valid (statistically significant), it is revealed in the GUHA output. Q-values were calculated by q-val package [21]. Statistical tests were two-sided at the 5% level of significance.

### Characterization of proteins

The spots generated from the statistical analyses as significantly changed were researched by their spot ID in the match set created by the PDQuest software. The relative molecular masses (Mr) and isoelectric points (pI) were estimated for each protein from their positions in the gels. The statistically important spots were considered for identification. The preparative 2-DE gels were prepared from cells with a relatively high content of the protein of interest using 400 μg of the cell lysate. They were stained with colloidal Coomassie stain [24].

### Mass spectrometry and protein identification

Selected spots on the preparative gels were excised and destained using 50% acetonitrile in 25 mM ammonium bicarbonate, dehydrated with 200 μl of acetonitrile for 5 min at 30°C using thermomixer comfort, Eppendorf AG (Hamburg, Germany) at 30°C and then vacuum-dried in SpeedVac, Thermo Scientific (Waltham, Ma). Gel pieces were rehydrated and proteins were digested for 8 hours at 37°C in the thermomixer with 30 ng/μl trypsin (Trypsin Gold Mass Spectrometry Grade, Promega, Madison, WI) in 25 mM ammonium bicarbonate. After digestion, peptides were extracted from gel pieces using step by step extraction with acetonitrile gradient (15%–60% acetonitrile with 1% trifluoroacetic acid). The extraction was performed in sonicator, Elma (Singen, Germany) with ice cubes.

Extracted peptides were concentrated in SpeedVac, Thermo Scientific (Waltham, Ma). Tandem electrospray ionization mass spectrometry (ESI-MS/MS) was used to characterize the digests. The ESI-MS/MS was performed in a quadrupole-time of flight (Q-TOF) tandem Micro mass spectrometer (Waters-Micromass) equipped with nanoelectrospray source and coupled to 2-D capillary chromatography CapLC (Waters). Chromatographic separation was achieved using the symmetry 300 Å OPTI-PAC (1 cm × 5 μm) trap column (Waters) and Atlantis dC18 (75 μm × 10 cm × 3 μm) capillary column (Waters). Data were processed by proteomic software Proteinlynx global server 2.1 (Waters) (LC-MS/MS).

## Results

### Clinico-pathological characteristics of the patients

**Figure 1:**
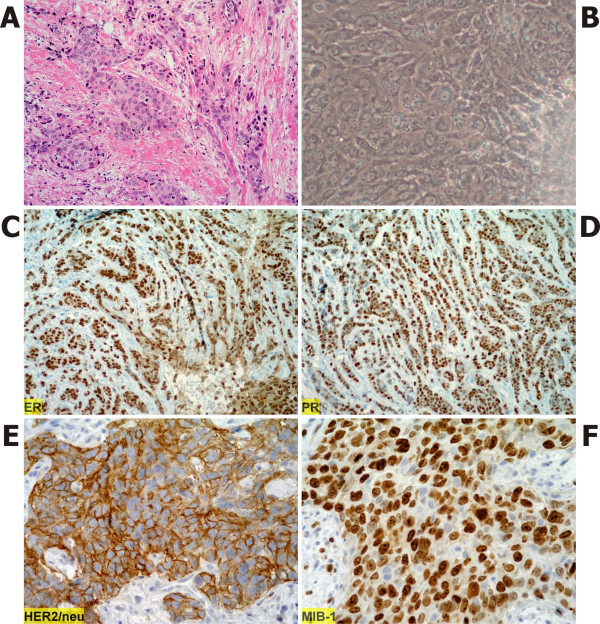
**Examples of positive staining of tumoral tissue sections**. A) Invasive ductal carcinoma, patient L116. Hematoxylin-eosin stain in 200× magnification reveals poor differentiation with a predominantly solid infiltrative pattern. The nuclei are highly pleomorphic with notable nucleoli. B) Outgrowths of cells from tumor fragment *in vitro *phase contrast (400× magnified). C) Immunostaining for ER, patient L116 (100× magnified). D) Immunostaining for PR, patient L116 (100× magnified). E) Immunostaining for HER 2/neu, patient L116 (400× magnified). F) Immunostaining for Ki67 with MIB-1 antibody, patient L122 (400× magnified).

Primary cultures of breast epithelial cells available for 2-DE analysis were cultivated from biopsy specimens of 23 breast cancer patients. An example of ER, PR and HER 2/neu positive immunocytochemical staining of tumor tissue of patient L116 together with *in vitro *outgrowths of the cells are shown in Figure 1(A–E). The example of positive staining for Ki67 with MIB-1 in Figure 1(F) is shown for sample L122. Dominating histology of tumors were invasive ductal carcinomas (16 patients), mostly high grade, five lobular carcinomas, one of them pleomorphic, one mucinous and one medulary carcinoma. Sixteen tumors were ER-positive, seven were ER-negative including three carcinomas, which were triple-negative (ER-negative, PR-negative, HER 2/neu negative). Four tumors were stained as HER 2/neu 3+ and five other were HER 2/neu 2+. The main tumor characteristics are given in Table 1. Metastatic spreading was proven in seven patients during postoperative follow-up. The pattern of metastases and time to relapse are given in Table 2.

**Table 1:** Clinico-pathological characteristics of patients

**Patient**	**Age**	**Stage**	**Histology**	**GRADE**	**ER%**	**PR%**	**HER2**	**MIB-1%**
L101	33	T2N2M0	invasive ductal	3	70	30	NEG	30
L103	48	T1N0M0	invasive ductal	2	85	100	3+	10
L106	36	T1N0M0	invasive ductal	3	70	90	NEG	20
L116	54	T1N1M0	invasive ductal	3	90	90	3+	10
**L118**	75	T4N1M0	invasive ductal	3	NEG	NEG	3+	20
L122	75	T2N0M0	invasive ductal	3	NEG	NEG	NEG	80
**L128**	44	T1N0M0	invasive ductal	3	90	20	NEG	20
L133	81	T4N0M0	invasive ductal	2	80	60	2+	5–10
L154	70	T1N0M0	invasive ductal	3	80	70	NEG	30
L176	74	T2N1M0	invasive ductal	3	NEG	NEG	2+	35
**L180**	77	T4N1M0	invasive ductal	2	100	70–80	3+	10–20
L181	63	T1N0M0	invasive ductal	1	75	NEG	NEG	35
**L190**	49	T3N1M0	invasive ductal	2	65	5	2+	20
L42	51	T4N1M0	invasive ductal	3	70	70	NEG	<5
**L53**	69	T4N0M0	invasive ductal	3	NEG	NEG	NEG	30
**L67**	61	T4N0M0	invasive ductal	3	NEG	NEG	NEG	30
L143	68	T1N0M0	invasive lobular		20	30	1+	<5
L187	57	T2N1M0	invasive lobular		90	NEG	NEG	10
**L40**	52	T4N2M0	invasive lobular		NEG	NEG	2+	10
L43	51	T2N1M0	invasive lobular		30	70	NEG	<5
L174	29	T1N0M0	medullary	3	NEG	<5	NEG	70
L179	73	T1N0M0	mucinous	2	100	90	2+	5
L50	52	T2N1M0	tubulolobular		30	NEG	NEG	30

The age of patients, stage in the TNM classification, histology, grade of tumors and HER 2/neu (HER2), estrogen receptor (ER), progesterone receptor (PR), and nuclear protein Ki67 (MIB-1) percentage of immunostained cells are listed for each patient. The patients constituting the metastase positive group are in bold.

**Table 2:** Metastatic pattern of patients in the metastase positive group

Patient	Age (years)	Time to relapse (month)	Metastases			
			Lung	Bone	Liver	Soft tissue
L118	75	3.9				yes
L128	44	32.2	yes	yes	yes	yes
L180	77	11.8	yes	yes		
L190	49	4.0				yes
L40	52	12.8	yes		yes	
L53	69	17.7		yes	yes	
L67	61	26.5				yes

We intended to find differences in protein expression between the samples from metastase-positive and metastase-negative patient groups.

### 2-DE protein separation and image analysis

**Figure 2:**
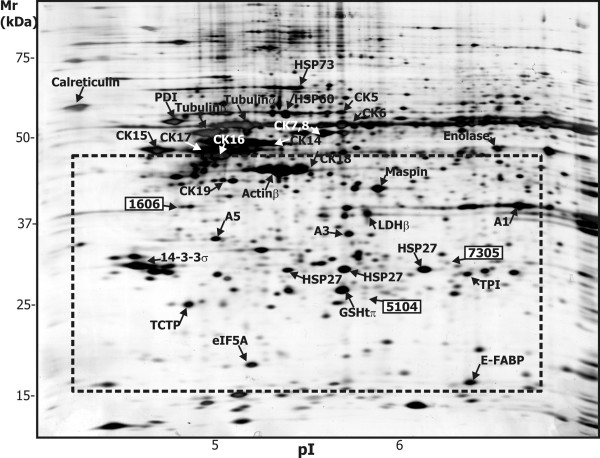
**Representative 2-DE map**. Representative 2-DE map of proteins of primary culture of epithelial breast cancer cells (sample L116). The gels were silver-stained. Solubilized proteins were focused on IPG strips (pH 4–7) and separated in SDS polyacrylamide gradient gels (8–16%). The characteristic proteins are named according to [15], A = annexin, CK = cytokeratin, eIF = eukaryotic translation factor, FABP = fatty acid-binding protein, GST = glutathione transferase, HSP = heat-shock protein, PDI = protein disulfide isomerase, TCTP = translationally controled tumor protein, TPI = triosephosphate isomerase. Protein spots significantly changed between the metastase-positive and metastase-negative samples are marked and described in Tables 3, 4, 5. The area of gel analyzed using PDQuest is outlined (------).

2-DE protein maps in the pH range 4–7 were prepared in triplicate. The 2-DE protein maps were very similar in all the cases and contained all the major spots that were identified in the normal mammary epithelial (NME) cell sample as described in [15]. A typical 2-DE gel is shown in Figure 2. The main protein spots are marked. The gels were analyzed by PDQuest Advanced 8.0.1 2D Gel Analysis Software. The software is not designed to compare multiple groups of samples that arise from our experimental setting (23 samples in triplicate). We compromised between the quantity of data and our ability to process them. The spot patterns in the area of the gels above Mr 50 kDa were not homogeneous and were not suitable for spot detection and matching by the PDQuest software due to huge protein clusters formed by cytokeratins and inconsistent streaks. Only spots in the pH range from 4.2 to 6.8 and Mr range from 15 kDa to 40 kDa were analyzed, matched and quantified. The area of the gel analyzed by PDQuest is outlined in Figure 2. A match-set containing 44 gels was created. Twenty-one samples were represented by two gels. Two samples were represented for capacity and gel quality reasons by one gel only. The correlation coefficients between technical replicates of gels ranged from 0.76 to 0.88. Matching of the most typical spots was checked and corrected manually. The spot patterns in distinct areas of gels were studied and spots unique to individual gels were added to the master gel to allow their analysis. Finally, 245 spots were matched and 70 of them were matched to all gels. The log-normalized spot quantity table containing all matched spots was generated and submitted to statistical analyses (Additional file 1).

### Statistical analysis and data mining

We searched for protein spots changed between the seven samples of metastase-positive and sixteen samples of metastase-negative groups.

For the GUHA analysis we averaged the intensity values for each sample. Categorization of 245 spot variables was determined on the basis of specification of maximal impurity decrease generating cut points. The basic task was to search for statistically significant relations between the presence/absence of spots and their classifications (metastatic/non-metastatic). In GUHA task settings, the antecedent variables were spot variables, succedent variables clinic variables. The lengths of antecedent and succedent were set to 1 and Fisher quantifier was employed. We searched for hypotheses rejecting the null hypothesis on independence between the presence/absence of spots and classification of 2-DE gels. Tests were taken at the 0.05 probability level. However, because we performed many simultaneous tests we had to adjust the significance level using the q-value. The q-value was calculated from the value of corresponding Fisher statistics. Q-values represent the approximation of maximal false-positive rate among the proteins supposed to be significant. Spots 7305 and 1606 fulfilled the criteria of q-value ≤ 0.05. The data from the GUHA analysis are presented in Table 3. We are showing 10 spots with the lowest q-values. The complete data can be found in Additional file 2.

**Table 3:** GUHA data mining analysis

Spot	Metastases	Cut point	Fisher	q-value
**7305**	**0**	**4.1**	**0.000069**	**0.0164**
**1606**	**1**	**67.6**	**0.000490**	**0.0370**
**5104**	**0**	**2.2**	**0.003953**	**0.0852**
4503	0	0.8	0.003953	0.0852
4103	0	2.4	0.003953	0.0852
1502	0	2.9	0.023960	0.1637
1002	0	3.1	0.025731	0.1637
4302	0	5.4	0.045004	0.2342
1001	0	19.3	0.045004	0.2342
8105	0	3.4	0.083004	0.2342

For each spot we show the number given by PDQuest software, hypotheses tested in GUHA (metastases = 1, no metastases = 0), cut points estimated from log-normalized density with maximal impurity function decrease, the value of Fisher statistics corresponding to Fisher's exact test (fisher) and maximal false-positive rate approximation among the proteins supposed to be significant (q-value).

**Figure 3:**
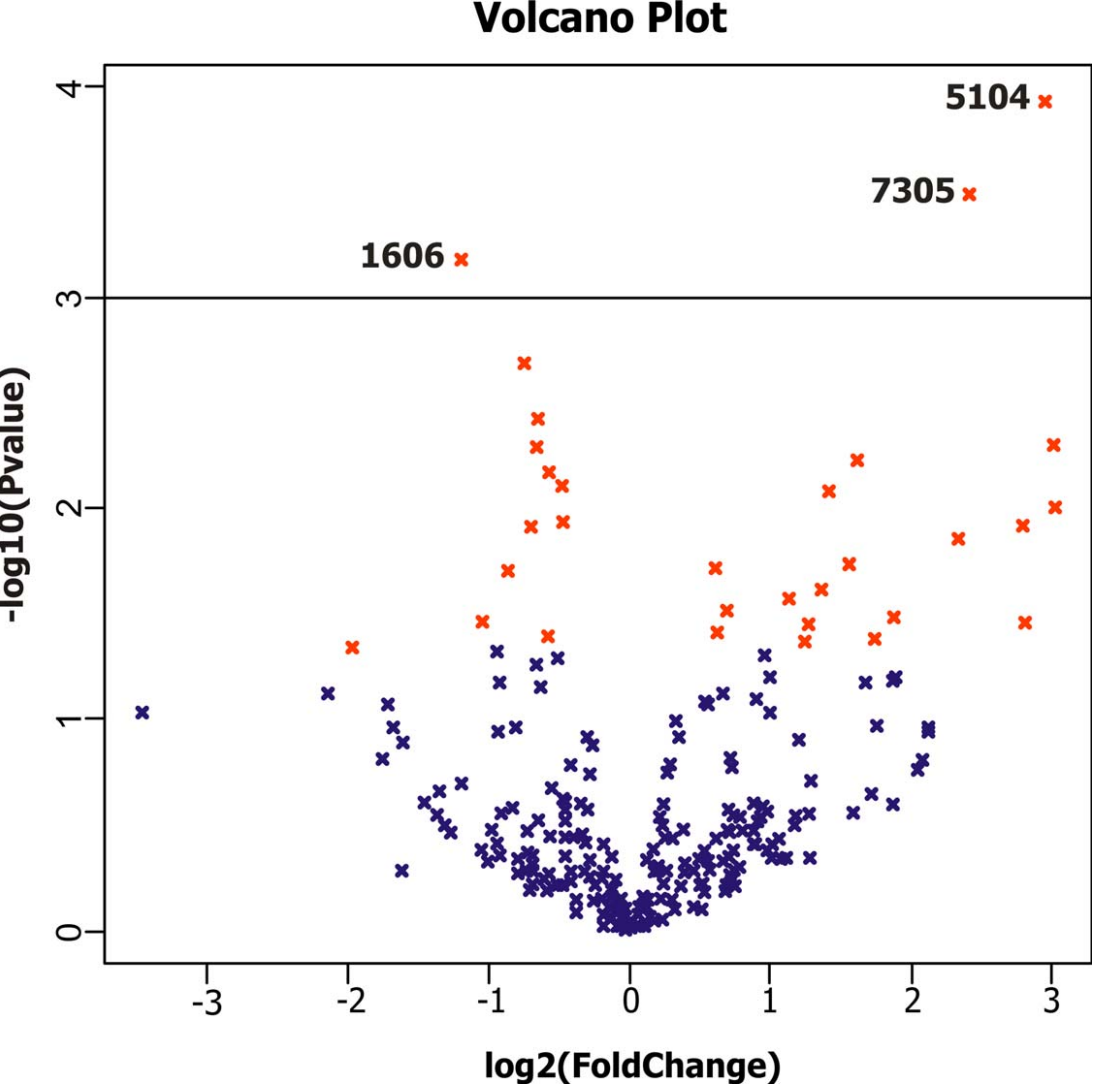
**Volcano plot**. The dots represent the individual spots from 2-DE analysis. The y-axis value is -log10(P-value) for the Fs test and the x-axis value is proportional to the fold changes (Huy, maanova [19]). The horizontal line represents the significance threshold 0.05 (Fs-adjusted). The red dots are the spots selected by the Fs test. Significantly changed spots are marked.

The false discovery rate adjusted p-value and the fold change were calculated using the R/maanova package [19]. All spots are presented in volcano plot (Huy) with log 2 fold change on x-axis and -log 10 (adjusted P-value) as y-axis (Figure 3). Spots 7305 and 5104 fulfilled statistical criteria of adjusted p-value ≤ 0.05 and log2 fold change ≥ 2 estimated by maanova. The data from maanova analysis are presented in Table 4. We are showing 10 spots with the lowest p-values and log2 fold change ≥ 1. The complete data can be found in Additional file 3.

**Table 4:** R/maanova

Spot	Fold change (log2)	Fs Pvalperm	Fs adjPvalperm
**5104**	**2.948184844**	**0.000152284**	**0.020651332**
**7305**	**2.401019096**	**0.000203046**	**0.020651332**
**1606**	**-1.190211206**	**0.000456853**	**0.030976998**
1502	3.018100581	0.002538071	0.086047217
8105	1.613027626	0.003959391	0.100675244
4302	1.414239041	0.005482234	0.100873814
1002	2.772977926	0.006395939	0.100873814
5301	3.016324495	0.006446701	0.100873814
4503	2.329847113	0.008629442	0.116809097
4103	1.547766562	0,013248731	0.150633246

For each spot we show the number given by PDQuest software, log2 difference of log-transformed means between metastases and non-metastases groups (fold change), nominal permutation p-values using Fs test statistics (Fs Pvalperm) and false discovery rate-adjusted p-values for multiple testing (Fs adjPvalperm).

We considered the spots 7305, 5104 and 1606 that have been found by either type of computing as significantly changed in correlation with the metastases. The spots 5104 and 7305 were negatively correlated with the metastatic phenotype, the spot 1606 was correlated positively.

### Characterization of proteins

**Figure 4:**
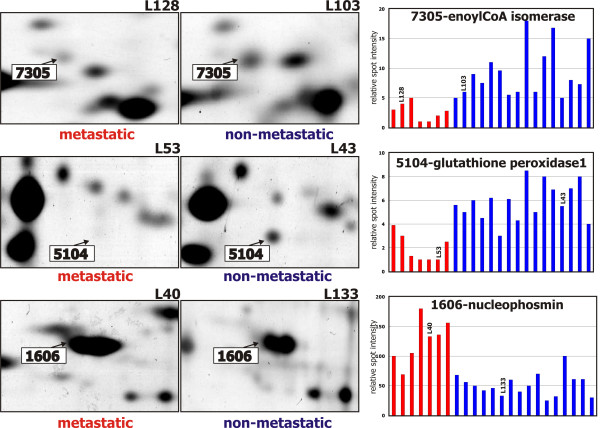
**Enlarged sections of representative 2-DE gels**. Enlarged sections of representative 2-DE gels showing differential expression of spots 7305, 5104 and 1606 between the metastic and non-metastic groups. The averaged relative intensities of particular spots in individual samples from the PDQuest output are shown in the bar graphs. The bars corresponding to the representative samples are marked. Sample order in the bar graph is the same as in Table 1 and in Table 2, the patient groups being separated.

The spots found by the statistical analyses were re-searched in the match-set created by the PDQuest software. The spots are marked in Figure 2 and enlarged sections of representative gels showing the respective spots in the metastatic and non-metastatic groups are presented in Figure 4. The data from protein identification are presented in Table 5. The identity of all peptides has been confirmed at least by three fragment ions in the MS/MS spectrum.

**Table 5:** Identification of significantly changed proteins between the metastase-positive and metastase-negative groups

Spot	Swiss-Prot protein name	Measured Mr/pI	Theor. Mr/pI	Sequence coverage (%)	Peptides sequenced	Score
7305	P42126	27/6.3	28/6	17	47–61 VLVEPDAGAGVAVMK	103
	2,3-trans-Enoyl-CoA				191–200 DTLENTIGHR	48.6
	Isomerase				271–283 DADVQNFVSFISK	18
					289–296 SLQMYLER	20.4
5104	P07203	22/5.9	21/6.1	10	147–155 LITWSPVC*R	5.4
	Glutathione peroxidase 1				165–175 FLVGPDGVPLR	12.1
1606	P06748	37/4.7	33/4.6	26	33–45 VDNDENEHQLSLR	53.6
	Nucleophosmin				55–73 DELHIVEAEAMNYEGSPIK	37.5
					81–101 MSVQPTVSLGGFEITPPVVLR	65.8
					238–246 GPSSVEDIK	20
					276–289 MTDQEAIQDLWQWR	24.9

For each spot we show the number given by PDQuest software, the accession number to the Swiss-Prot database, name, experimentally found (measured) and theoretical (theor.) relative molecular masses in kDa (Mr) and isoelectric points (pI), achieved sequence coverage (%), peptides sequenced by the LC-MS/MS, their composition, position and peptide score. C*: modified cystein detected as carbamidomethyl

Spot 1606 had a diffused pattern in the 2-DE gel. It was identified as nucleophosmin, [Swiss-Prot:P06748]. Spots 7305 and 5104 were of very low intensity as could be estimated from the cut-off values and judged from Figures 2 and 4. Spot 7305 was identified as 2,3-trans-enoyl-CoA isomerase, [Swiss-Prot:P42126]. Spot 5104 was identified as glutathione peroxidase 1, [Swiss-Prot:P07203].

## Discussion

Breast cancer is one of the most intensively studied cancers. However, the breast cancer research proved to be extremely complicated due to the complex biology of mammary gland [11,14]. We believe that our method of temporal *in vitro *propagation of cells from breast cancer tissues [12,17] could partially lead to the selection of cells relatively close to putative tumor stem/progenitor cells. We suppose that through analysis of these cells we might indicate proteins responsible for the overall tumor behavior. We performed the 2-DE analysis of 23 primary cultures of epithelial cells derived from breast cancer tissues from which seven samples were metastase-positive.

All the 2-DE gels from different samples were similar to each other and were conformable to the normal mammary epithelial (NME) cell sample described in Selicharova et al [15]. The similarity of individual primary cultures of breast cancer cells was a good prerequisite for performing the comparative proteomic experiment. On the other hand, the experimental design based on the primary cultures substantially decreased the available amount of samples necessary for powerful statistics. From 120 human breast tumors we only obtained 23 usable cell cultures with a sufficient amount of cultured cells (about five millions).

Only few spots apparently varied among sets of gels from individual samples qualitatively or quantitatively. Some of the proteins have been identified (data not shown). We detected variations in quantity of cathepsin D, cathepsin B, squamous cell carcinoma antigen, γ synuclein, cytokeratin 19 and other proteins that have been reported to play a role in cancer etiology [25-29]. We also found isoelectric variants of several proteins arising from common polymorphism that might have impact on the cancerogenesis (glutathione transferase ω, glyoxalase I) [30,31]. On the other hand we have not observed variation in quantity of heat shock proteins (HSP 90, HSP 60, HSP 27) or the molecular chaperone 14-3-3 σ among our breast cancer cell cultures. These proteins have been reported to be altered in the breast cancer [5,32]. Although these findings were exciting, we could not demonstrate without further validation the significance of above mentioned observations and their connectedness with the tumor characteristics.

We intended to perform computational quantitative analysis of our 2-DE data. Any computer software designed to align and compare 2-DE gels must somehow deal with distorted spot patterns that are pertinent to the methodology. So far, the spot detection and matching must be supervised by a researcher [10] which was another bottleneck of our experimental design. We have analyzed our gels by the PDQuest Advanced 8.0.1 2D Gel Analysis Software since it is available in our laboratory. The software is not designed to compare multiple groups of samples that have arisen from our experimental setting (23 samples in triplicate). We compromised between the quantity of data and our ability to process them. We have finally constructed a match set composed of 44 cropped gels yielding well distinguished spot patterns. The spot patterns were carefully studied and matching was adjusted in each gel. Finally, 245 spots were matched and their normalized quantities in each gel were subjected to statistical analyses and data mining. The quantification of proteins in 2-DE gels is relative and it is a matter of dynamic range versus sensitivity [8,24]. The gels were silver-stained because we intended to achieve the utmost sensitivity to be able to detect possible changes in the expression of less abundant proteins in our samples. We are aware that this type of staining might be a source of inaccuracy. The correlation coefficients between technical replicates of gels ranged from 0.76 to 0.88 which is normal for the 2-DE analyses [9]. However, the overall variability within the data cut down the attainable statistical significance of our results. In spite of all the disputable issues we indicated spots correlated with metastases in the set of patients. We used two different statistical approaches to search for significant correlations between clinical data and spot intensities. The GUHA [20] enriched with cut-off points and q-values computes with categorical variables sorted according to the cut points. The R/maanova [19] computes with integers that correspond to the spot densities. The outputs of the methods slightly differ but in general the same spots were found with both the methods as significantly correlated with the clinical data.

Spots 7305, 5104 and 1606 fulfilled the statistical criteria in either analysis. Spot 7305 was identified as 2,3-trans-enoyl-CoA isomerase. The enzyme is involved in mitochondrial β-oxidation of unsaturated fatty acids [33]. The defects in distribution of polyunsaturated fatty acids in healthy and cancerous breast tissues have been documented [34]. Decreased levels of this enzyme might have impact on the aberrant behavior of cancer cells.

Spot 5104 was identified as glutathione peroxidase 1, a selenium dependent enzyme that detoxifies hydrogen and lipid peroxides. The protective function of selenium against cancer mortality has been documented. It remains unclear how selenium decreases cancer risk and whether glutathione peroxidase is involved in the action [35]. The lowered levels of the enzyme in our group of patients with metastases further support possible involvement of the glutathione peroxidase 1 in the anticancer defense.

Spot 1606 was increased in the group of patients with metastases and it appeared to be abundant. The spot had a diffused pattern in the 2-DE gel and the MS spectra were complicated. It was identified as nucleophosmin, a highly phosphorylated protein associated with nucleolar ribonucleoprotein structures [36]. The protein is known to be extensively post-translationally modified. It might be a reason for its 2-DE pattern but we cannot exclude that there might be other proteins contained in the spot. Nucleophosmin is overexpressed in many types of human solid tumors. It is a multifunctional protein and its physiological function in tumorigenesis is controversial [37].

## Conclusion

We have performed an extensive proteomic study of mammary epithelial cells from the breast cancer patients. We found three proteins that were significantly altered in the group of patients who developed distant metastases within the three-year post-operative follow-up. After development of preferably an immunocytochemical detection methodology, our results need to be proven on a higher amount of samples (directly on tumor biopsies) to establish their value for prediction of tumor behavior, spreading or as potential treatment targets. It should be kept in mind that we were able to process only a small part of the 2-DE maps. Potentially, more proteins may be found if we analyze other portions of the proteomes of our samples. The work is in progress.

## List of abbreviations

2-DE: two-dimensional gel electrophoresis; ER: estrogen receptor; ESI: electrospray ionization; GUHA: General Unary Hypothesis Automaton; HER 2/neu: human epidermal growth factor receptor 2; LC-MS/MS: liquid chromatography-tandem mass spectrometry; Q-TOF: quadrupole-time of flight; PR: progesterone receptor

## Competing interests

The author(s) declare that they have no competing interests.

## Authors' contributions

JV designed and coordinated the study, evaluated and interpreted the clinical data, contributed to statistical computations and drafted the manuscript. IS designed and performed the analysis of 2-DE gels, prepared the samples for identification of proteins, analyzed, evaluated and interpreted the proteomical data and drafted the manuscript. KS optimized the methodology for preparation of 2-DE gels and prepared the gels. MS performed the LC-MS/MS experiments and protein identifications. EM contributed to the study concept and design, was responsible for cell cultures establishment and handling and contributed to writing of the manuscript. EB and MP established and handled the cell cultures. ZV performed the imunohistochemistry analysis. DC was responsible for the GUHA and statistical analyses and contributed to writing of the manuscript. JJ contributed to the study concept and design and critically revised the manuscript for important intellectual content. All authors read and approved the final manuscript.

## Pre-publication history

The pre-publication history for this paper can be accessed here:



## Supplementary Material

Additional file 1 **PDQuest quantity table**. The table is in the excel spreadsheet format (PDQuest.xls). Spots are numbered in rows. Means of logarithmic ratios method normalized spot densities in each gel are in columns. The paired gels from each sample (named according to the Table 1) are marked (a) respective (b), for example L40-sp-a, L40-sp-b. Click here for file

Additional file 2 **Results of the GUHA analysis**. The table is in the excel spreadsheet format (guha.xls). Hypotheses are numbered in rows. Antecedent column displays categorized spot intensities. Succedent column displays presence (= 1) or absence (= 0) of metastases at follow-up. Table column displays the contingency table evaluating cedents. Fisher quantifier column corresponds to Fisher's exact test. Click here for file

Additional file 3 **Results of the R/maanova analysis**. The table is in the excel spreadsheet format (maanova.xls). For each spot we show in adequate columns the number given by PDQuest software, log2 difference of log transformed means between metastases and non-metastases groups (fold change), nominal permutation p-values using F1 test statistics (F1 Pvalperm) and false discovery rate adjusted p-values for multiple testing (F1 adjPvalperm). Nominal permutation p-values using Fs test statistics (Fs Pvalperm) and false discovery rate-adjusted p-values for multiple testing (Fs adjPvalperm). Click here for file
